# Optimal Design of Hierarchical Cloud-Fog&Edge Computing Networks with Caching

**DOI:** 10.3390/s20061582

**Published:** 2020-03-12

**Authors:** Xiaoqian Fan, Haina Zheng, Ruihong Jiang, Jinyu Zhang

**Affiliations:** 1 School of Computer and Information Technology, Beijing Jiaotong University, Beijing 100044, China; xqfan1995@bjtu.edu.cn (X.F.); rhjiang@bjtu.edu.cn (R.J.); zjy@bjtu.edu.cn (J.Z.); 2 State Key Lab of Rail Traffic Control and Safety, Beijing Jiaotong University, Beijing 100044, China

**Keywords:** fog&edge computing, cloud computing, content caching, computation offloading, energy minimization

## Abstract

This paper investigates the optimal design of a hierarchical cloud-fog&edge computing (FEC) network, which consists of three tiers, i.e., the cloud tier, the fog&edge tier, and the device tier. The device in the device tier processes its task via three computing modes, i.e., cache-assisted computing mode, cloud-assisted computing mode, and joint device-fog&edge computing mode. Specifically, the task corresponds to being completed via the content caching in the FEC tier, the computation offloading to the cloud tier, and the joint computing in the fog&edge and device tier, respectively. For such a system, an energy minimization problem is formulated by jointly optimizing the computing mode selection, the local computing ratio, the computation frequency, and the transmit power, while guaranteeing multiple system constraints, including the task completion deadline time, the achievable computation capability, and the achievable transmit power threshold. Since the problem is a mixed integer nonlinear programming problem, which is hard to solve with known standard methods, it is decomposed into three subproblems, and the optimal solution to each subproblem is derived. Then, an efficient optimal caching, cloud, and joint computing (CCJ) algorithm to solve the primary problem is proposed. Simulation results show that the system performance achieved by our proposed optimal design outperforms that achieved by the benchmark schemes. Moreover, the smaller the achievable transmit power threshold of the device, the more energy is saved. Besides, with the increment of the data size of the task, the lesser is the local computing ratio.

## 1. Introduction

### 1.1. Background

With the rapid development of wireless communications technologies and the wide deployment of mass smart devices, a large number of emerging applications [1], such as artificial intelligence (AI) [2], augmented reality (AR) [3], and virtual reality (VR) [4], have been arising in Internet of Things (IoT) networks, which put forward higher requirements for computation capability and transmit power to the smart device in the IoT network [5]. As we all know, most smart devices usually have limited communication, computation, storage, and energy resources, which is a huge challenge to complete such computation-intensive and delay-sensitive applications [6,7,8,9,10].

To solve these problems, fog&edge computing (FEC) is regarded as a potential solution via providing computing service to smart devices on the edge of the network, which meets the requirements of the smart device for processing the computation-intensive and delay-sensitive tasks in real time [11,12,13]. The FEC has two advantages: (i) Compared with local computing by the device itself [14], FEC enables the limited computation capabilities of the smart devices. (ii) Compared with cloud computing [15], FEC reduces the delay caused by long distances and traffic congestion for offloading to the cloud server.

Apart from computation offloading in the FEC, content caching is another promising technology to solve the limited computation capability of the device and reduce the transmission delay [16]. Significantly, an important part of the delay is caused via the redundant transmission and computation of a few popular files, such as the files for rendering scenes in the typical VR application scenario [17]. Therefore, caching popular content in the fog&edge tier is an effective way to avoid duplicate transmission and computation.

To face the challenge of completing the computation-intensive and delay-sensitive applications, integrating three such technologies, i.e., cloud computing, fog&edge computing, and content caching, into a single network system could bring strong performance improvement, which is of great significance.

### 1.2. Related Work

Over the past few years, a large number of research works has investigated cloud computing [18,19,20], fog&edge computing [21,22,23,24,25,26,27,28,29], and content caching [30,31,32]. However, most of these works involved these three technologies separately.

Then, the combination of two technologies began to be studied; see, e.g., [13,21,22,33,34,35,36,37,38,39,40,41]. Specifically, in [33], the authors investigated an energy efficiency maximization problem in a cloud-assisted FEC system. In [34], the authors proposed a cloud-assisted mobile edge computing (MEC) framework designed to guarantee user service quality with minimal system cost. In [35], the authors investigated a heterogeneous cloud-MEC two-tier offloading framework, and an computing offloading scheme was designed to minimize overall energy consumption. In [36], the authors proposed an integration framework of the cloud, MEC, and IoT to solve the scalability problem of MEC and designed a selective offloading scheme to achieve the minimum energy consumption of mobile devices while meeting the delay requirements. In [37], the authors investigated the optimal workload allocation problem in a fog-cloud computing system toward the minimal power consumption with constrained service delay. However, non of the above works involved content caching.

On the other hand, in [21], the authors studied joint service caching and task offloading for MEC-enabled dense cellular networks. In [22], the authors proposed a collaborative offloading scheme to cache the popular computation results to reduce the task execution delay. In [38], the authors investigated an optimization problem that considered offloading decisions, computing resources, and content caching. An alternative direction algorithm based on a multiplier was proposed to solve the maximize revenue problem. In [39], the authors proposed a joint caching and offloading mechanism to minimize the average total energy minimization problem. In [40,41], the authors investigated an energy minimization problem for a cache-aided FEC system. However, none of the above works involved cloud computing.

Recently, a few works began to study these three technologies in a single system to further improve system performance, i.e., in [42], the offloading and caching strategy was studied for a cloud-assisted FEC system to minimize delay, where however, it was not the aim to reduce the energy consumption. In [43], the offloading and caching decision was investigated for a hybrid cloud/edge computing system to minimize energy consumption, where however, only the binary decision was considered.

### 1.3. Motivation and Contributions

As mentioned above, there exist a few works that have studied these three technologies together, and to the best of our knowledge, no work has been done on the optimal design of a hierarchical cloud-FEC network with caching to minimize the energy consumption. Therefore, to explore the benefits of cloud computing, fog&edge computing, and content caching, we study the optimal design of a hierarchical cloud-FEC network with caching to minimize the energy consumption.

The main contributions of our work are summarized as follows.

- A three-tier network framework is considered, and correspondingly, we propose three computing modes to process the computation task of the device, i.e., cache-assisted computing mode, cloud-assisted computing mode, and joint device-fog&edge computing mode. Specifically, the task corresponds to being completed via the content caching in the FEC tier, the computation offloading to the cloud tier, and the joint computing in the fog&edge and device tier, respectively.
- For such a system, an energy minimization problem is formulated by jointly optimizing the computing mode selection, the local computing ratio, the computation frequency, and the transmit power, while guaranteeing multiple system constraints, including the task completion deadline time, the achievable computation capability, and the achievable transmit power threshold.
- Since the problem is a mixed integer nonlinear programming problem, which is hard to solve with known standard methods, it is decomposed into three subproblems, and the optimal solution to each subproblem is derived. Then, an efficient optimal caching, cloud, and joint computing (CCJ) algorithm to solve the primary problem is proposed.
- Simulation results show that the system performance achieved by our proposed optimal design outperforms that achieved by the benchmark schemes. Moreover, the smaller the achievable transmit power threshold of the device, the more energy is saved. Besides, with the increment of the data size of the task, the lesser is the local computing ratio.

The rest of this paper is organized as follows. Section 2 describes the system model, and the optimization problem is formulated. In Section 3, the closed-form and semi-closed-form solutions to the three subproblems are derived, and an efficient algorithm, i.e., the CCJ algorithm, is presented. Section 4 provides some simulation results, and finally, Section 5 summarizes this paper.

## 2. System Model and Problem Formulation

### 2.1. System Model

**Figure 1:**
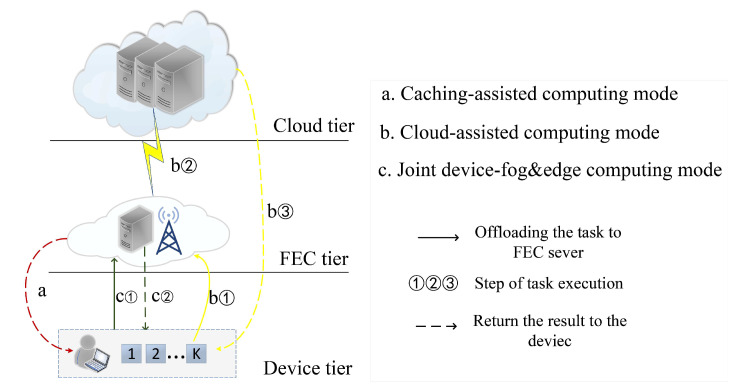
Illustration of the hierarchical cloud-fog&edge computing network.

Consider a hierarchical cloud-FEC network as shown in Figure 1, which consists of three tiers, i.e., the cloud tier, the fog&edge tier, and the device tier. Specifically, in the device tier, the smart device generates *K* different computation tasks, which follow the uniform distribution of K, where K denotes the set of task types with K≜{1,...,K}. Each task is computed locally or offloaded. In the FEC tier, the FEC server is deployed at the base station (BS), which has the content caching and the computation capability to process the offloaded tasks. Besides, the BS is connected to the cloud server in the cloud tier via optical fiber. For any task *k*, for example, face recognition is a typical application scenario, which usually consists of five main computing components, including image acquisition, face detection, preprocessing, feature extraction, and classification. Image acquisition components can be executed on devices to support the user interface, but other complex computing components, such as signal processing and the machine learning (ML) algorithm, can be offloaded to the fog&edge computing or cloud computing to execute. Some of the components are cached in the FEC server in advance, which could reduce computing delay and energy consumption of the device.

We define task *k* as $\tau_{k}≜\left\{a_{k},C_{k},d_{k},T_{k}^{\max}\right\}$, where $a_{k}\in \left\{0,1\right\}$ is the caching indicator. When $a_{k}=1$, it indicates that the kth task has been cached in the FEC tier, and when $a_{k}=0$, it indicates that the kth task has not been cached. $C_{k}$ is the number of central processing unit (CPU) cycles required for computing one bit of the kth task; $d_{k}$ is the data size of the kth task; and $T_{k}^{\max}$ is the completion deadline time of the kth task.

For the FEC tier, due to limited caching space, we assume that the FEC server only caches several of the most popular files. The popularity of the files follows a Zipf distribution. Therefore, the popularity of the kth task is described as:

(1) $$\begin{array}{c} z_{k}=\frac{\frac{1}{k^{\mu }}}{\sum_{k=1}^{K}\frac{1}{k^{\mu }}}, \end{array}$$

where μ is the shape parameter and is regarded as constant [44,45]. For our considered system, denote *Z* as the caching threshold according to the popularity. When $z_{k}\geq Z$, the kth task is cached; otherwise, the task is not cached.

**Figure 2:**
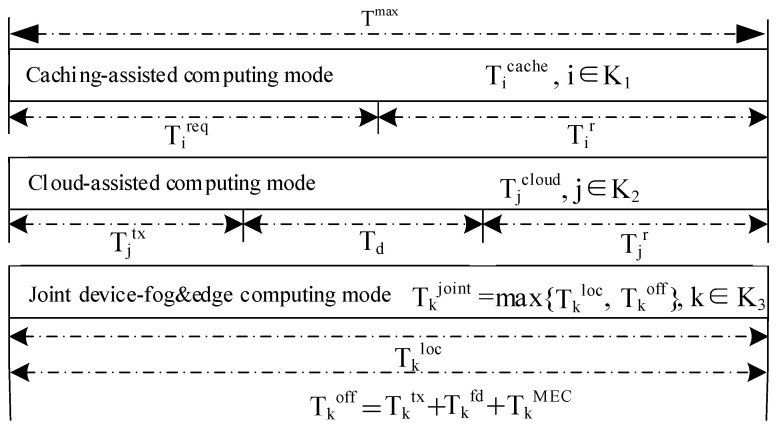
Illustration of the transmit protocol for a given time block.

For each task, the transmit protocol is shown in Figure 2. When the task is cached, it is processed in the caching computing mode.

#### 2.1.1. Caching-Assisted Computing Mode

For the caching-assisted computing mode, the delay includes two parts. One is the task request time $T_{k}^{\mathrm{req}}$, which is too small to be ignored. The other is the result feedback time $T_{k}^{r}$, which depends on the data size of the results. The delay of task *k* in the caching-assisted computing mode is given by:

(2) $$\begin{array}{c} T_{k}^{\mathrm{cache}}=T_{k}^{\mathrm{req}}+T_{k}^{r}\approx \frac{\delta d_{k}}{B\log_{2}\left(1+\frac{p^{\mathrm{FEC}}{\left|h\right|}^{2}}{\sigma^{2}}\right)}. \end{array}$$

where δ is the data ratio of the results. ${\left|h\right|}^{2}$, $p^{\mathrm{FEC}}$, $f^{\mathrm{FEC}}$, *B*, and $\sigma^{2}$ are the channel coefficient between the device and the FEC tier, the transmit power and the computing capability of the FEC server, and the system bandwidth and the noise power, respectively. The energy consumption of device for task *k* in the caching-assisted computing mode is given by:

(3) $$\begin{array}{c} E_{k}^{\mathrm{cache}}=p^{c}T_{k}^{\mathrm{cache}}, \end{array}$$

where $p^{c}$ is the circuit power of the device for waiting.

When the task is not cached, it is processed in the joint device-fog&edge computing mode or the cloud-assisted computing mode. Furthermore, we define $\gamma_{k}\in \left\{0,1\right\}$ as the uncached task execution decision, where $\gamma_{k}=1$ indicates that the cloud-assisted computing mode is selected; otherwise, the joint device-fog&edge computing mode is selected.

#### 2.1.2. Cloud-Assisted Computing Mode

Consider a cloud tier with a strong enough computation capability, so the execute time in the cloud tier can be neglected. For the cloud-assisted computing mode, the delay includes three parts. One is the task transmission time between the device and the FEC tier $T_{k}^{\mathrm{ts}}$. One is the task transmission time between the FEC tier and the cloud tier $T_{d}$, which depends on the distance between the FEC tier and the cloud tier and is regarded as a constant in this work. The other one is the result feedback time $T_{k}^{r}$. Therefore, the total delay of task *k* is given by:

(4) $$\begin{array}{cc} T_{k}^{\mathrm{cloud}}= & T_{k}^{\mathrm{tx}}+T_{d}+T_{k}^{r}=\frac{d_{k}}{B\log_{2}\left(1+\frac{p_{k}^{\mathrm{tx}}{\left|h\right|}^{2}}{\sigma^{2}}\right)}+T_{d}+\frac{\delta d_{k}}{B\log_{2}\left(1+\frac{p^{\mathrm{FEC}}{\left|h\right|}^{2}}{\sigma^{2}}\right)}. \end{array}$$

Meanwhile, the energy consumption of the device for task *k* is given by:

(5) $$\begin{array}{c} E_{k}^{\mathrm{cloud}}=p_{k}^{\mathrm{tx}}T_{k}^{\mathrm{tx}}+p^{c}T_{k}^{\mathrm{cloud}}, \end{array}$$

where $p_{k}^{\mathrm{tx}}$ is the transmit power of the device for task *k*.

#### 2.1.3. Joint Device-Fog&Edge Computing Mode

For joint device-fog&edge computing mode, the device portions each task into two parts. One part executes by local computing. The other one executes by offloading to the FEC tier for computing.

- Local execution
  According to most existing related works, to achieve minimal energy consumption, an identical CPU frequency should be adopted for each CPU cycle. Thus, we denote $f_{k}^{\mathrm{loc}}$ as the average computation frequency of the device for each bit of the kth task. Therefore, the execution time of task *k* is given by:
  (6) $$\begin{array}{c} T_{k}^{\mathrm{loc}}=\frac{\beta_{k}C_{k}d_{k}}{f_{k}^{\mathrm{loc}}}, \end{array}$$
  where $\beta_{k}\in \left[0,1\right]$ is the ratio of task *k* for local execution at the device and $\left(1-\beta_{k}\right)$ represents the offloading ratio of task *k* for FEC execution.
  The energy consumption of the device for task *k* is given by:
  (7) $$E_{k}^{\mathrm{loc}}=\kappa f_{k}^{\mathrm{loc}3}T_{k}^{\mathrm{loc}}=\kappa f_{k}^{\mathrm{loc}2}\beta_{k}C_{k}d_{k},$$
  where κ is the effective switched capacitor depending on the chip architecture.
- FEC execution
  The FEC execution delay includes three parts. The first one is task offloading time $T_{k}^{\mathrm{tx}}$. The last one is FEC execution time $T_{k}^{\mathrm{FEC}}$. The other is the result feedback time $T_{k}^{\mathrm{fd}}$. Thus, the delay of FEC execution for task *k* is given by:
  (8) $$\begin{array}{cc} T_{k}^{\mathrm{off}} & =T_{k}^{\mathrm{tx}}+T_{k}^{\mathrm{FEC}}+T_{k}^{\mathrm{fd}}\\ & =\frac{\left(1-\beta_{k}\right)d_{k}}{B\log_{2}\left(1+\frac{p_{k}^{\mathrm{tx}}{\left|h\right|}^{2}}{\sigma^{2}}\right)}+\frac{\left(1-\beta_{k}\right)C_{k}d_{k}}{f^{\mathrm{FEC}}}+\frac{\delta \left(1-\beta_{k}\right)d_{k}}{B\log_{2}\left(1+\frac{p^{\mathrm{FEC}}{\left|h\right|}^{2}}{\sigma^{2}}\right)}. \end{array}$$
  The energy consumption of the device in FEC execution for task *k* is given by:
  (9) $$\begin{array}{c} E_{k}^{\mathrm{off}}=p_{k}^{\mathrm{tx}}T_{k}^{\mathrm{tx}}+p^{c}T_{k}^{\mathrm{off}}. \end{array}$$

As a result, the total delay of task *k* in the joint device-fog&edge computing mode is:

(10) $$\begin{array}{c} T_{k}^{\mathrm{joint}}=\max\left\{T_{k}^{\mathrm{loc}},T_{k}^{\mathrm{off}}\right\}, \end{array}$$

and the energy consumption of the device for task *k* in this mode is given by:

(11) $$\begin{array}{c} E_{k}^{\mathrm{joint}}=E_{k}^{\mathrm{loc}}+E_{k}^{\mathrm{off}}. \end{array}$$

Denote $K_{1}$, $K_{2}$, $K_{3}$ as the set in the caching-assisted computing, cloud-assisted computing, and joint device-fog&edge computing mode, respectively. $|K_{1}|$, $|K_{2}|$, and $|K_{3}|$ are the element number of $K_{1}$, $K_{2}$, and $K_{3}$, respectively. Then, the average energy consumption of the device is given by:

(12) $$\begin{array}{cc} E^{\mathrm{ave}} & =\frac{\sum_{k}^{K_{1}}E_{k}^{\mathrm{cache}}+\gamma_{k}\sum_{k}^{K_{2},K_{3}}E_{k}^{\mathrm{cloud}}+\left(1-\gamma_{k}\right)\sum_{k}^{K_{2},K_{3}}E_{k}^{\mathrm{joint}}}{|K_{1}\left|+\right|K_{2}\left|+\right|K_{3}|},K_{1}\cup K_{2}\cup K_{3}=K. \end{array}$$

### 2.2. Problem Formulation

Our goal is to minimize the average energy consumption of the device in the hierarchical cloud-FEC system. Mathematically, the average energy minimization problem is formulated as:

(13) $$P_{0}:\min_{\beta ,\gamma ,p^{\mathrm{tx}},f^{\mathrm{loc}}}\;E^{\mathrm{ave}}$$

(13a) $$s.t.\;\;T_{i}^{\mathrm{cache}}\leq T_{i}^{\max},\forall i\in K_{1},$$

(13b) $$\;\;\;T_{j}^{\mathrm{cloud}}\leq T_{j}^{\max},\forall j\in K_{2},$$

(13c) $$\;\;\;T_{k}^{\mathrm{joint}}\leq T_{k}^{\max},\forall k\in K_{3},$$

(13d) $$\;\;\;0\leq f_{k}^{\mathrm{loc}}\leq f^{\max},\forall k\in K_{1},K_{2},K_{3},$$

(13e) $$\;\;\;\;0\leq p_{k}^{\mathrm{tx}}\leq p^{\max},\forall k\in K_{1},K_{2},K_{3},$$

(13f) $$\;\;\;\;\beta_{k}\in \left[0,1\right],\forall k\in K_{3},$$

(13g) $$\;\;\;\gamma_{k}\in \left\{0,1\right\},\forall k\in K_{2},K_{3},$$

where $\beta ≜{\left[\beta_{1},\beta_{2},\ldots ,\beta_{K}\right]}^{T}$, $\gamma ≜{\left[\gamma_{1},\gamma_{2},\ldots ,\gamma_{K}\right]}^{T}$, $p^{\mathrm{tx}}≜{\left[p_{1}^{\mathrm{tx}},p_{2}^{\mathrm{tx}},\ldots ,p_{K}^{\mathrm{tx}}\right]}^{T}$, and $f^{\mathrm{loc}}≜{\left[f_{1}^{\mathrm{loc}},f_{2}^{\mathrm{loc}},\ldots ,f_{K}^{\mathrm{loc}}\right]}^{T}$ denote the local computing ratio, the computing mode selection, the transmit power, and the computation frequency of the device, respectively. $f^{\max}$ and $p^{\max}$ denote the maximal achievable computation frequency and transmit power of the device, respectively. Constraints (13a), (13b), and (13c) mean that the delay in the three computing modes cannot exceed the completion deadline time, respectively. Constraints (13d) and (13e) represent the computation capability constraint and transmit power of the device, respectively.

## 3. Optimal Solution Approach

In this section, in order to solve Problem $P_{0}$, we shall first decompose it into three subproblems. Then, by respectively solving them, the optimal solution to Problem $P_{0}$ is derived.

### 3.1. Optimization of the Caching-Assisted Computing Mode

As mentioned above, when $z_{i}\geq Z$, $i\in K_{1}$ and the caching-assisted computing mode is employed. In this mode, the optimal energy consumption is:

(14) $$\begin{array}{cc} E_{i}^{\mathrm{cache}}= & p^{c}T_{i}^{r}=\frac{p^{c}\delta d_{i}}{B\log_{2}\left(1+\frac{p^{\mathrm{FEC}}{\left|h\right|}^{2}}{\sigma^{2}}\right)}. \end{array}$$

**Proposition** **1.** *When $p^{FEC}$ has its maximal achievable threshold, $E_{i}^{cache}$ achieves the optimal value.*

**Proof** **of** **Proposition** **1.** The larger $p^{\mathrm{FEC}}$ is, the smaller $T_{i}^{r}$ is, and the smaller $E_{i}^{\mathrm{cache}}$ is. Therefore, when $p^{\mathrm{FEC}}$ is with its maximal achievable threshold, the energy consumption of the device reaches its minimum value. Therefore, the optimal $E_{i}^{\mathrm{cache}}$ can be obtained. Thus, Proposition 1 is proven. □

### 3.2. Optimization of the Cloud-Assisted Computing Mode

When $z_{j}<Z$ and $\gamma_{j}=1$, $j\in K_{2}$ and the cloud-assisted computing mode is employed. In this mode, the optimal problem is expressed as:

(15) $$P_{1}:\min_{p_{j}^{\mathrm{tx}}}\;\;E_{j}^{\mathrm{cloud}}$$

(15a) $$s.t.\;T_{j}^{\mathrm{cloud}}\leq T_{j}^{\max},$$

(15b) $$\;\;\;\;0\leq p_{j}^{\mathrm{tx}}\leq p^{\max}.$$

By expanding the expressions of the variables of Problem $P_{1}$, it is equivalently rewritten as:

(16) $$P_{1\_A}:\min_{p_{j}^{\mathrm{tx}}}\;\;p^{c}\left(\frac{d_{j}}{B\log_{2}\left(1+\frac{p_{j}^{\mathrm{tx}}{\left|h\right|}^{2}}{\sigma^{2}}\right)}+T_{d}+\frac{\delta d_{j}}{B\log_{2}\left(1+\frac{p^{\mathrm{FEC}}{\left|h\right|}^{2}}{\sigma^{2}}\right)}\right)+p_{j}^{\mathrm{tx}}\frac{d_{j}}{B\log_{2}\left(1+\frac{p_{j}^{\mathrm{tx}}{\left|h\right|}^{2}}{\sigma^{2}}\right)}$$

(16a) $$s.t.\;\;\frac{d_{j}}{B\log_{2}\left(1+\frac{p_{j}^{\mathrm{tx}}{\left|h\right|}^{2}}{\sigma^{2}}\right)}\;+T_{d}+\;\frac{\delta d_{k}}{B\log_{2}\left(1+\frac{p^{\mathrm{FEC}}{\left|h\right|}^{2}}{\sigma^{2}}\right)}\;\;\leq T_{j}^{\max},\;\;\;\;$$

(16b) $$0\leq p_{j}^{\mathrm{tx}}\leq p^{\max}.$$

**Lemma** **1.** *Problem $P_{1\_A}$ is a convex optimization problem.*

**Proof** **of** **Lemma** **1.** Denote $f\left(p_{j}^{\mathrm{tx}}\right)=p^{c}\left(\frac{d_{j}}{B\log_{2}\left(1+\frac{p_{j}^{\mathrm{tx}}{\left|h\right|}^{2}}{\sigma^{2}}\right)}+T_{d}+\frac{\delta d_{j}}{B\log_{2}\left(1+\frac{p^{\mathrm{FEC}}{\left|h\right|}^{2}}{\sigma^{2}}\right)}\right)$. The second order derivative of $f(p_{j}^{\mathrm{tx}})$ is always larger than zero, so the objective function of Problem $P_{1\_A}$ is convex. The first constraint is rewritten as $g(p_{j}^{\mathrm{tx}})\leq 0$, i.e., $\frac{d_{j}}{B\log_{2}\left(1+\frac{p_{j}^{\mathrm{tx}}{\left|h\right|}^{2}}{\sigma^{2}}\right)}+\frac{\delta d_{j}}{B\log_{2}\left(1+\frac{p^{\mathrm{FEC}}{\left|h\right|}^{2}}{\sigma^{2}}\right)}+T_{d}-T_{j}^{\max}\leq 0$, and its second order derivative is also always larger than zero. Therefore, the first constraint is also convex. Therefore, Problem $P_{1\_A}$ is a convex optimization problem. Lemma 1 is proven. □

With Lemma 1 and the derivative of $g(p_{j}^{\mathrm{tx}})=0$, the optimal solution to Problem $P_{1\_A}$ is that $p_{j}^{\mathrm{tx}*}=\min\left\{\frac{\left(Np^{c}-1\right)W\left(0,exp\left(-1\right)\left(Np^{c}-1\right)\right)-1}{N},p^{\max}\right\}$, where $N=\frac{{\left|h\right|}^{2}}{\sigma^{2}}$.

### 3.3. Optimization of the Joint Device-Fog&Edge Computing Mode

When $z_{k}<Z$ and $\gamma_{k}=0$, $k\in K_{3}$ and the joint device-fog&edge computing mode is employed. The optimization problem can be expressed as:

(17) $$P_{2}:\min_{\beta_{k},p_{k}^{\mathrm{tx}},f_{k}^{\mathrm{loc}}}\;\;E_{k}^{\mathrm{loc}}+E_{k}^{\mathrm{off}}$$

(17a) $$s.t.\;\;T_{k}^{\mathrm{joint}}\leq T_{k}^{\max},$$

(17b) $$\;\;\;\;0\leq f_{k}^{\mathrm{loc}}\leq f^{\max},$$

(17c) $$\;\;\;\;0\leq p_{k}^{\mathrm{tx}}\leq p^{\max},$$

(17d) $$\;\;\;\;\beta_{k}\in \left[0,1\right].$$

We design an alternating iteration method to solve Problem $P_{2}$. Firstly, we fix $\beta_{k}$, and the primal Problem $P_{2}$ becomes a sub-problem in terms of $p_{k}^{\mathrm{tx}}$ and $f_{k}^{\mathrm{loc}}$. Then, we substitute the optimal values of $p_{k}^{\mathrm{tx}}$ and $f_{k}^{\mathrm{loc}}$ into Problem $P_{2}$, and Problem $P_{2}$ is reformulated as a subproblem in terms of $\beta_{k}$. Hence, Problem $P_{2}$ is divided into two sub-problems as follows, i.e., Problem $P_{3}$ w.r.t. the transmit power $p_{k}^{\mathrm{tx}}$ and computation frequency $f_{k}^{\mathrm{loc}}$ and Problem $P_{4}$ w.r.t. the local computing ratio $\beta_{k}$.

Let $\beta_{k}^{\left(0\right)}$ be the feasible point to Problem $P_{2}$. Problem $P_{2}$ is re-expressed as:

(18) $$P_{3}:\min_{p_{k}^{\mathrm{tx}},f_{k}^{\mathrm{loc}}}\;\;E_{k}^{\mathrm{loc}}+E_{k}^{\mathrm{off}}$$

(18a) $$s.t.\;T_{k}^{\mathrm{loc}}\leq T_{k}^{\max},$$

(18b) $$\;\;\;T_{k}^{\mathrm{off}}\leq T_{k}^{\max},$$

(18c) $$\;\;\;0\leq f_{k}^{\mathrm{loc}}\leq f^{\max},$$

(18d) $$\;\;\;\;0\leq p_{k}^{\mathrm{tx}}\leq p^{\max}.$$

To solve Problem $P_{3}$, we expand the expressions of the variables of Problem $P_{3}$ to be:

(19) $$\begin{array}{c} P_{3\_A}:\min_{p_{k}^{\mathrm{tx}},f_{k}^{\mathrm{loc}}}\;\;\kappa f_{k}^{\mathrm{loc}2}\beta_{k}^{\left(0\right)}C_{k}d_{k}+\frac{p_{k}^{\mathrm{tx}}\left(1-\beta_{k}^{\left(0\right)}\right)d_{k}}{B\log_{2}\left(1+\frac{p_{k}^{\mathrm{tx}}{\left|h\right|}^{2}}{\sigma^{2}}\right)}+p^{c}(\frac{\left(1-\beta_{k}^{\left(0\right)}\right)d_{k}}{B\log_{2}\left(1+\frac{p_{k}^{\mathrm{tx}}{\left|h\right|}^{2}}{\sigma^{2}}\right)}\\ +\frac{\left(1-\beta_{k}^{\left(0\right)}\right)C_{k}d_{k}}{f^{\mathrm{FEC}}}+\frac{\delta \left(1-\beta_{k}^{\left(0\right)}\right)d_{k}}{B\log_{2}\left(1+\frac{p^{\mathrm{FEC}}{\left|h\right|}^{2}}{\sigma^{2}}\right)}) \end{array}$$

(19a) $$s.t.\;\;\frac{\beta_{k}^{\left(0\right)}C_{k}d_{k}}{f_{k}^{\mathrm{loc}}}\leq T_{k}^{\max},$$

(19b) $$\frac{\left(1-\beta_{k}^{\left(0\right)}\right)d_{k}}{B\log_{2}\left(1+\frac{p_{k}^{\mathrm{tx}}{\left|h\right|}^{2}}{\sigma^{2}}\right)}+\frac{\left(1-\beta_{k}^{\left(0\right)}\right)C_{k}d_{k}}{f^{\mathrm{FEC}}}+\frac{\delta \left(1-\beta_{k}^{\left(0\right)}\right)d_{k}}{B\log_{2}\left(1+\frac{p^{\mathrm{FEC}}{\left|h\right|}^{2}}{\sigma^{2}}\right)}\leq T_{k}^{\max},$$

(19c) $$\;\;\;\;0\leq f_{k}^{\mathrm{loc}}\leq f^{\max},$$

(19d) $$\;\;\;\;0\leq p_{k}^{\mathrm{tx}}\leq p^{\max}.$$

**Lemma** **2.** *Problem $P_{3\_A}$ is a convex optimization problem.*

**Proof** **of** **Lemma** **2.** Let the objective function as $\beta_{k}^{\left(0\right)}h_{1}\left(f_{k}^{\mathrm{loc}}\right)+\left(1-\beta_{k}^{\left(0\right)}\right)h_{2}\left(p_{k}^{\mathrm{tx}}\right)$, where $h_{1}\left(f_{k}^{\mathrm{loc}}\right)=\kappa f_{k}^{\mathrm{loc}2}C_{k}d_{k}$ and $h_{2}\left(p_{k}^{\mathrm{tx}}\right)=p_{k}^{\mathrm{tx}}\frac{d_{k}}{B\log_{2}\left(1+\frac{p_{k}^{\mathrm{tx}}{\left|h\right|}^{2}}{\sigma^{2}}\right)}+p^{c}\left(\frac{d_{k}}{B\log_{2}\left(1+\frac{p_{k}^{\mathrm{tx}}{\left|h\right|}^{2}}{\sigma^{2}}\right)}+\frac{C_{k}d_{k}}{f^{\mathrm{FEC}}}+\frac{\delta d_{k}}{B\log_{2}\left(1+\frac{p^{\mathrm{FEC}}{\left|h\right|}^{2}}{\sigma^{2}}\right)}\right)$. The second order derivatives of $h_{1}\left(f_{k}^{\mathrm{loc}}\right)$ and $h_{2}\left(p_{k}^{\mathrm{tx}}\right)$ are respectively given by:

(20) $$\begin{array}{c} \frac{\partial^{2}h_{1}\left(f_{k}^{\mathrm{loc}}\right)}{\partial f_{k}^{\mathrm{loc}2}}>0,\frac{\partial^{2}h_{2}\left(p_{k}^{\mathrm{tx}}\right)}{\partial p_{k}^{\mathrm{tx}2}}>0, \end{array}$$

which means that the objective function is convex. The first constraint is re-written as $\beta_{k}^{\left(0\right)}h_{3}\left(f_{k}^{\mathrm{loc}}\right)+\left(1-\beta_{k}^{\left(0\right)}\right)\left\{h_{4}\left(p_{k}^{\mathrm{tx}}\right)+\frac{d_{k}}{B\log_{2}\left(1+\frac{p_{k}^{\mathrm{tx}}{\left|h\right|}^{2}}{\sigma^{2}}\right)}+\frac{C_{k}d_{k}}{f^{\mathrm{FEC}}}+\frac{\delta d_{k}}{B\log_{2}\left(1+\frac{p^{\mathrm{FEC}}{\left|h\right|}^{2}}{\sigma^{2}}\right)}-T_{k}^{\max}\right\}\leq 0$. The second order derivatives of $h_{3}\left(f_{k}^{\mathrm{loc}}\right)$ and $h_{4}\left(p_{k}^{\mathrm{tx}}\right)$ are respectively derived as:

(21) $$\begin{array}{c} \frac{\partial^{2}h_{3}\left(f_{k}^{\mathrm{loc}}\right)}{\partial f_{k}^{\mathrm{loc}2}}>0,\frac{\partial^{2}h_{4}\left(p_{k}^{\mathrm{tx}}\right)}{\partial p_{k}^{\mathrm{tx}2}}>0. \end{array}$$

Therefore, the constraint is also convex. Lemma 2 is proven. □

Lemma 2 indicates that Problem $P_{3}$ is a joint convex optimization problem w.r.t. $f_{k}^{\mathrm{loc}}$ and $p_{k}^{\mathrm{tx}}$, which can be solved by using some standard convex optimization tools, such as CVX. According to Proposition 1, when $p^{\mathrm{FEC}}=p^{\max}$ and $f^{\mathrm{FEC}}=f^{\max}$, the optimal $E_{k}^{\mathrm{joint}}$ can be achieved.

By substituting the optimal solution of $f_{k}^{\mathrm{loc}}$ and $p_{k}^{\mathrm{tx}}$ into Problem $P_{3}$ to get $E_{k}^{\mathrm{loc}(0)}$ and $E_{k}^{\mathrm{off}(0)}$, we have:

(22) $$P_{4}:\min_{\beta_{k}}\;\;\beta_{k}E_{k}^{\mathrm{loc}(0)}+\left(1-\beta_{k}\right)E_{k}^{\mathrm{off}(0)}$$

(22a) $$s.t.\;T_{k}^{\mathrm{joint}}\leq T_{k}^{\max},$$

(22b) $$\;\;\;\;\beta_{k}\in \left[0,1\right].$$

Since the objective function of Problem $P_{4}$ is linear w.r.t. $\beta_{k}$, it is solved by several well-studied method.

**Algorithm 1** Optimal caching, cloud, and joint computing (CCJ) algorithm.
1:**Initialize**$C_{i},d_{i},p^{c},K_{1}=0,K_{2}=0,K_{3}=0$, and other known parameters;2:
**for** i=1:|K| **do**
3:    **if** $a_{k}=1$ **then**4:        Calculate $E_{k}^{\mathrm{cache}}$ according to (14);5:        $k\to K_{1};$6:    **else**7:        Calculate $E_{k}^{\mathrm{cloud}}$ according to (15);8:        Calculate $E_{k}^{\mathrm{joint}}$ according to (17);9:        **if** $E_{k}^{\mathrm{cloud}}<E_{k}^{\mathrm{joint}}$ **then**10:            $\gamma_{k}=1;$11:            $k\to K_{2};$12:        **else**13:            $\gamma_{k}=0;$14:            $k\to K_{3};$15:        **end** **if**16:    **end** **if**17:
**end** **for**
18:Calculate $\sum_{i}^{K_{1}}E_{i}^{\mathrm{cache}}$, $\sum_{j}^{K_{2}}E_{j}^{\mathrm{cloud}}$, $\sum_{i}^{K_{3}}E_{k}^{\mathrm{joint}}$19:Calculate $E^{\mathrm{ave}}$ according to (13);


With the closed-form or well-structured solutions to the cloud-assisted computing mode and the joint device-fog&edge computing mode in Section 3.2 and Section 3.3, the minimal energy consumption (i.e., $E_{j}^{\mathrm{cloud}}$ and $E_{k}^{\mathrm{joint}}$) can be calculated. Therefore, for uncached task *k*, $\forall k\in K_{2},K_{3}$, the computing mode selection can be determined by:

$$\gamma_{k}=\left\{\begin{array}{cc} 0, & \mathrm{if}\;E_{k}^{\mathrm{cloud}}>\;E_{k}^{\mathrm{joint}},\\ 1, & \mathrm{otherwise}. \end{array}\right.$$

In order to show our proposed algorithm clearly, i.e, the optimal caching, cloud, and joint computing (CCJ) algorithm, we summarize it as shown in Algorithm 1. It is able to converge to the global optimal solution with low computational complexity.

## 4. Simulation Results

### 4.1. Simulation Setup

In this section, we present some numerical results to discuss the performance of the hierarchical cloud-FEC system. We considered a centralized FEC network covered by a 200 m × 200 m area, where the BS was connected to the cloud server via optical fiber. In the device tier, the number of tasks requested by the device was K=10. The input data size of the task was randomly distributed within [100,1000] MB, and the data ratio of the result δ was 0.1. The corresponding number of required CPU cycles was distributed within [0.2,1] G-cycles. The maximum achievable transmit power of the device was set as $p^{\max}$ = 0.1 W. The circuit power of the device was $p^{c}$ = 0.01 W. In the FEC tier, the maximum achievable transmit power and computation capability of the FEC server was $p^{\mathrm{FEC}}$ = 1 W and $f^{\mathrm{FEC}}=5$ G-cycles, respectively. In the cloud tier, since the calculation delay in the cloud server was ignored, we set $T_{d}$ = 0.2 s, which was the transmission delay regarding the distance between the FEC tier and the cloud tier. In terms of communication, the system bandwidth was set as *B* = 3 MHz, and the white Gaussian noise was set to be $\sigma^{2}=10^{-8}$ W [29]. In addition, the channel gain was modeled by h=127+30×logd with independent Rayleigh fading, where *d* is the distance between the device and the FEC server. According to the realistic measurements in [46], we set the effective switched capacitor $\kappa =10^{-26}$. In the caching policy, we set the shape parameter μ = 0.56 and the caching threshold *Z* = 0.16. In this paper, all experiments were implemented in MathWorks MATLAB R2016b on a laptop equipped with a 12.00 GHz Corei5-3337U CPU and 128 GB random access memory. Every point in the figures was the result averaged over $10^{4}$ independent channel realizations.

We compared our proposed algorithm with three different benchmark schemes as follows:

- No caching (NC) scheme: This scheme supposed that the FEC system did not have a cache function. Therefore, the task could only be executed through cloud computing mode or joint computing mode.
- Caching and joint execution (CJE) scheme: This scheme used our proposed cache policy. For the uncached task, it could be processed by the joint computing mode, that is γ=0.
- Caching and cloud execution (CCE) scheme: This scheme used our proposed cache policy. For the uncached task, it could be processed by the cloud computing mode, that is γ=1.

### 4.2. Experimental Results

**Figure 3:**
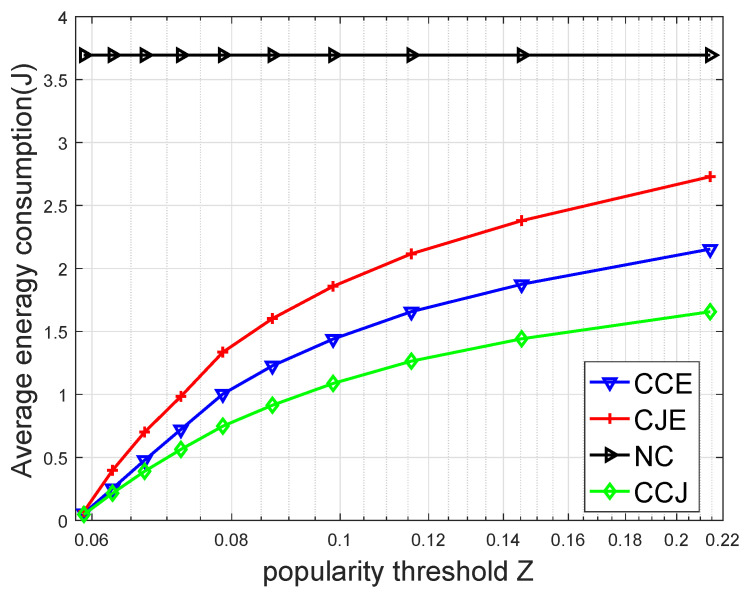
Average energy consumption on four schemes versus the caching popularity threshold.

Figure 3 compares the average energy consumption versus different caching popularity thresholds. It is seen that with the increment of *Z*, the average energy consumption of the device increased. The reason is that the larger the *Z*, the more the task was cached and processed in the FEC tier. The energy consumption was mainly caused by the circuit consumption of the device during waiting for the FEC server to execute the task and return the results to the device.

**Figure 4:**
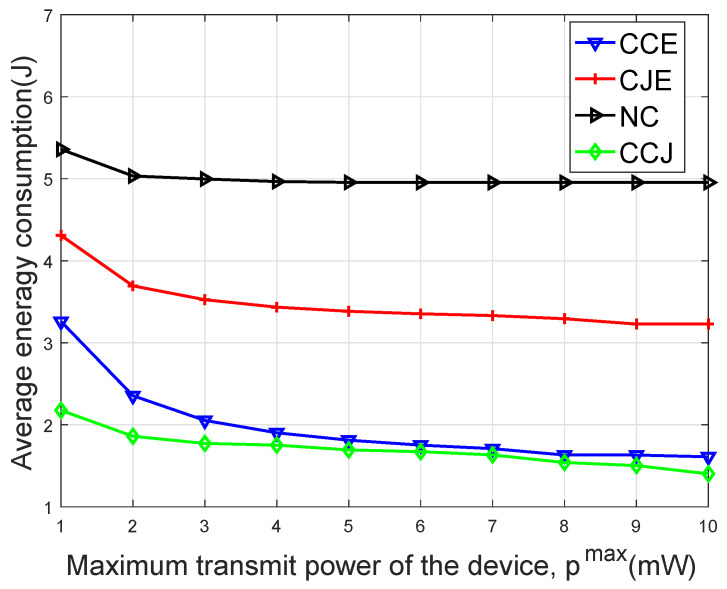
Average energy consumption of the four schemes versus the maximum achievable transmit power of device $p^{\max}$.

Figure 4 shows the average energy consumption versus different maximal achievable transmit powers of the device. It is seen that with $p^{\max}$ increasing, the average energy consumption of the device decreased and finally tended to be stable. The reason was that the higher the transmit power of the device, the faster the transmission rate, and the less the transmission time, the lower the energy consumption of the device. When $p^{\max}$ was relatively small, the optimal solution of the transmit power was on the boundary, i.e., $p^{\max}$. When $p^{\max}$ reached a certain value, the optimal solution of the transmit power shall not change.

**Figure 5:**
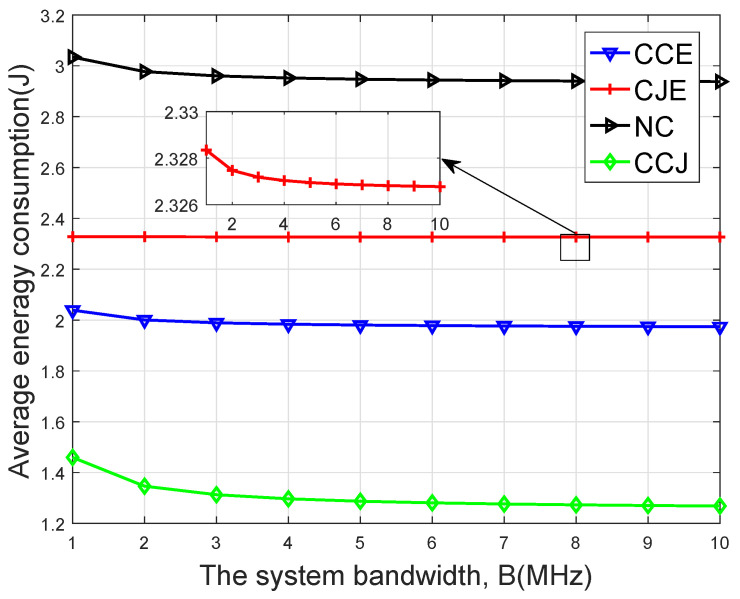
Average energy consumption on the four schemes versus the system bandwidth *B*.

Figure 5 compares the average energy consumption versus different system bandwidths. It is seen that with the bandwidth increasing, the average energy consumption decreased. The reason may be that the larger the system bandwidth, the larger the transmission rate, which resulted in less delay and lower energy consumption.

**Figure 6:**
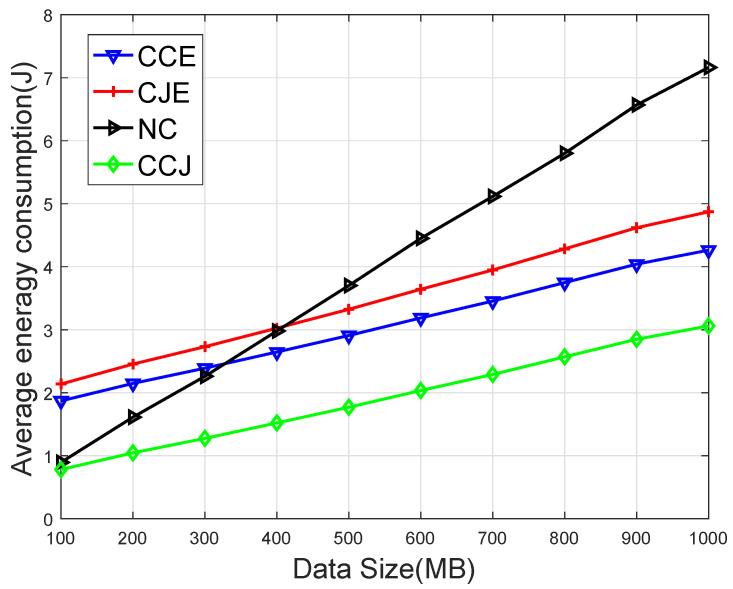
Average energy consumption on the four schemes versus the data size $d_{k}$.

Figure 6 compares the average energy consumption versus different task data sizes. It is seen that with the data size increasing, the average energy consumption increased. The reason may be that the larger the data size of the task, the larger the transmission delay and the calculation delay, which led to the greater energy consumption of the device.

**Figure 7:**
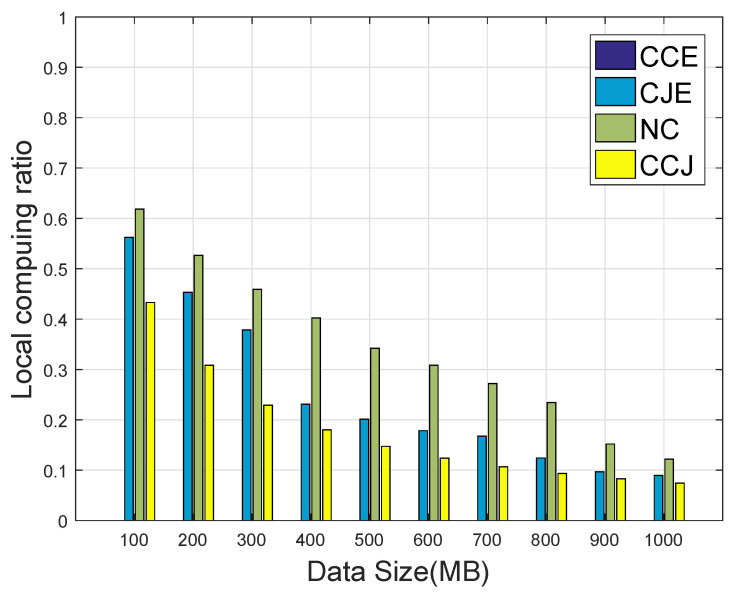
Average energy consumption on the four schemes versus the data size $d_{k}$.

Figure 7 compares the local computing ratio versus different task data sizes. It is seen that with the increment of the data size of the task, the local computing ratio decreased. The reason was that when the data size of the task was small, it was computed locally with less energy consumption compared with offloading. When the data size of the task was large, the computation capacity of the device was not enough to support the calculation, and more parts of the task should be offloaded to the FEC tier for computing.

## 5. Conclusions

This paper studied the optimal design of a hierarchical cloud-FEC network with caching. For such a system, an energy minimization problem was formulated by jointly optimizing the computing mode selection, the local computing ratio, the computation frequency, and the transmit power of the device, while guaranteeing multiple system constraints, including the task completion deadline time, the achievable computation capability, and the achievable transmit power threshold of the device.Since the problem was a mixed integer nonlinear programming problem, which was hard to solve, it was decomposed into three subproblems, and the optimal solution for each subproblem was derived. Then, an efficient CCJ algorithm to solve the primary problem was designed. Simulation results showed that the system performance achieved by our proposed optimal design outperformed that achieved by the benchmark schemes. Specifically, compared with the NC scheme, the energy consumption reduced by our proposed optimal design by about 56%. Compared with the CJE scheme, the energy consumption reduced by our proposed optimal design by about 44%. Compared with the CCE scheme, the energy consumption reduced by our proposed optimal design by about 5%. Moreover, the smaller the achievable transmit power threshold of the device, the more energy was saved. Besides, with the increment of the data size of the task, the lesser was the local computing ratio.

## Abbreviations

The following abbreviations are used in this manuscript:

IoT	Internet of Things
FEC	Fog&edge computing
AI	Artificial intelligence
ML	Machine learning
AR	Augmented reality
VR	Virtual reality
CPU	Central processing unit
BS	Base station
CCJ	Caching cloud joint
NC	No caching
CCE	Caching cloud execution
CJE	Caching joint execution

## References

[1] Qin M., Chen L., Zhao N., Chen Y., Yu F.R., Wei G. (2018). Power-constrained edge computing with maximum processing capacity for IoT networks. IEEE Internet Things J..

[2] Wang L., Jiao L., Li J., Gedeon J. (2018). Moera: Mobility-agnostic online resource allocation for edge computing. IEEE Trans. Mob. Comput..

[3] Dong Y., Guo S., Liu J., Yang Y. (2019). Energy-efficient fair cooperation fog computing in mobile edge networks for smart city. IEEE Internet Things J..

[4] Mehrabi A., Siekkinen M., Ylä-Jääski A. (2018). Edge computing assisted adaptive mobile video streaming. IEEE Trans. Mob. Comput..

[5] (2017). Cisco Visual Networking Index: Global Mobile Data Traffic Forecast Update.

[6] Wang T., Lu Y., Cao Z., Lei S., Zheng X., Liu A., Xie M. (2019). When Sensor-Cloud Meets Mobile Edge Computing. Sensors.

[7] Zheng H., Xiong K., Fan P., Zhou L., Zhong Z. (2018). SWIPT-aware fog information processing: Local computing vs. fog offloading. Sensors.

[8] Mao Y., You C., Zhang J., Huang K., Letaief K. (2017). A survey on mobile edge computing: the communication perspective. IEEE Commun. Surv. Tutor..

[9] Jeong H.J. Lightweight Offloading System for Mobile Edge Computing. Proceedings of the IEEE PerCom Workshops.

[10] Xiong K., Chen C., Qu G., Fan P., Letaief K.B. (2017). Group cooperation with optimal resource allocation in wireless powered communication networks. IEEE Trans. Wirel. Commun..

[11] Cui T., Hu Y., Shen B., Chen Q. (2019). Task Offloading Based on Lyapunov Optimization for MEC-Assisted Vehicular Platooning Networks. Sensors.

[12] Wang P., Yao C., Zheng Z., Sun G., Song L. (2019). Joint task assignment, transmission, and computing resource allocation in multilayer mobile edge computing systems. IEEE Internet Things J..

[13] Ren J., Yu G., Yu G., He Y., Li G.Y. (2019). Collaborative cloud and edge computing for latency minimization. IEEE Trans. Veh. Technol..

[14] Neto J.L.D., Yu S.Y., Macedo D.F., Nogueira J.M.S., Langar R., Secci S. (2018). ULOOF: A user level online offloading framework for mobile edge computing. IEEE Trans. Mob. Comput..

[15] Mian G., Li L., Guan Q. (2019). Energy-efficient and delay-guaranteed workload allocation in IoT-edge-cloud computing systems. IEEE Access.

[16] Wei H., Luo H., Sun Y. (2020). Mobility-Aware Service Caching in Mobile Edge Computing for Internet of Things. Sensors.

[17] Liu X., Sun C., Zhang X. Context-aware caching with social behavior in MEC-enabled wireless cellular networks. Proceedings of the IEEE PerCom Workshops.

[18] Zhou B., Dastjerdi A.V., Calheiros R.N., Srirama S.N., Buyya R. (2017). Mcloud: A context-aware offloading framework for heterogeneous mobile cloud. IEEE Trans. Serv. Comput..

[19] Mahmoodi S.E., Uma R.N., Subbalakshmi K.P. (2019). Optimal joint scheduling and cloud offloading for mobile applications. IEEE Trans. Cloud Comput..

[20] Misra S., Wolfinger B.E., Achuthananda M.P., Chakraborty T., Das S.N., Das S. (2019). Auction-Based Optimal Task Offloading in Mobile Cloud Computing. IEEE Syst. J..

[21] Xu J., Chen L., Zhou P. Joint service caching and task offloading for mobile edge computing in dense networks. Proceedings of the IEEE INFOCOM.

[22] Yu S., Langar R., Fu X., Wang L., Han Z. (2018). Computation offloading with data caching enhancement for mobile edge computing. IEEE Trans. Veh. Technol..

[23] Hu G., Jia Y., Chen Z. Multi-user computation offloading with d2d for mobile edge computing. Proceedings of the IEEE GLOBECOM.

[24] Wang Y., Sheng M., Wang X., Wang L., Li J. (2016). Mobile-edge computing: partial computation offloading using dynamic voltage scaling. IEEE Trans. Commun..

[25] Guo H., Liu J., Zhang J. (2018). Computation offloading for multi-access mobile edge computing in ultra-dense networks. IEEE Internet Things J..

[26] Guo H., Liu J. (2018). Collaborative Mobile-Edge Computation Offloading for IoT over Fiber-Wireless Networks. IEEE Network.

[27] Rodrigues T.G., Suto K., Nishiyama H., Kato N. (2017). Hybrid Method for Minimizing Service Delay in Edge Cloud Computing Through VM Migration and Transmission Power Control. IEEE Trans. Comput..

[28] Liu M., Liu Y. (2017). Price-based distributed offloading for mobile-edge computing with computation capacity constraints. IEEE Commun. Lett..

[29] Hao Y., Chen M., Hu L., Hossain M.S., Ghoneim A. (2018). Energy efficient task caching and offloading for mobile edge computing. IEEE Access.

[30] Hou T., Feng G., Qin S., Jiang W. Proactive Content Caching by Exploiting Transfer Learning for Mobile Edge Computing. Proceedings of the IEEE Globecom.

[31] Jia G., Han G., Du J., Chan S. (2018). A maximum cache value policy in hybrid memory-based edge computing for mobile devices. IEEE Internet Things J..

[32] Ale L., Zhang N., Wu H., Chen D., Han T. (2019). Online proactive caching in mobile edge computing using bidirectional deep recurrent neural network. IEEE Internet Things J..

[33] Tao X., Ota K., Dong M., Qi H., Li K. (2017). Performance guaranteed computation offloading for mobile-edge cloud computing. IEEE Commun. Lett..

[34] Ma X., Zhang S., Yang P., Lin C., Shen X.S. Cost-Efficient Resource Provisioning in Cloud Assisted Mobile Edge Computing. Proceedings of the IEEE Globecom.

[35] Dai Y., Xu D., Maharjan S., Zhang Y. (2018). Joint computation offloading and user association in multi-task mobile edge computing. IEEE Trans. Veh. Technol..

[36] Lyu X., Tian H., Jiang L., Vinel A., Maharjan S., Gjessing S., Zhang Y. (2018). Selective offloading in mobile edge computing for the green internet of things. IEEE Netw..

[37] Deng R., Lu R., Lai C., Luan T.H., Liang H. (2016). Optimal workload allocation in fog-cloud computing toward balanced delay and power consumption. IEEE Internet Things J..

[38] Wang C., Liang C., Chen Q., Tang L. Joint computation offloading, resource allocation and content caching in cellular networks with mobile edge computing. Proceedings of the IEEE ICC.

[39] Cui Y., He W., Ni C., Guo C., Liu Z. Energy-efficient resource allocation for cache-assisted mobile edge computing. Proceedings of the IEEE LCN.

[40] Pietro D., Strinati E.C. (2019). An optimal low-complexity policy for cache-aided computation offloading. IEEE Access.

[41] Liu P., Xu G., Yang K., Wang K., Meng X. (2019). Jointly optimized energy-minimal resource allocation in cache-enhanced mobile edge computing systems. IEEE Access.

[42] Zhang J., Hu X., Ning Z., Ngai E., Zhou L., Wei J., Cheng J., Hu B., Leung V.C.M. (2018). Joint resource allocation for latency-sensitive services over mobile edge computing networks with caching. IEEE Internet Things J..

[43] Yang X., Fei Z., Zheng J., Zhang N., Anpalagan A. (2019). Joint multi-user computation offloading and data caching for hybrid mobile cloud/edge computing. IEEE Trans. Veh. Technol..

[44] Wang C., Liang C., Yu F.R., Chen Q., Tang L. (2017). Computation offloading and resource allocation in wireless cellular networks with mobile edge computing. IEEE Trans. Wireless Commun..

[45] Breslau L., Cao P., Fan L., Phillips G., Shenker S. Web caching and Zipf-like distributions: Evidence and implications. Proceedings of the IEEE INFOCOM.

[46] Rappaport T.S. (1996). Wireless Communications: Principles and Practice.

